# Influence of Host and Environmental Factors on the Distribution of the Japanese Encephalitis Vector *Culex tritaeniorhynchus* in China

**DOI:** 10.3390/ijerph15091848

**Published:** 2018-08-27

**Authors:** Boyang Liu, Xiang Gao, Jun Ma, Zhihui Jiao, Jianhua Xiao, Hongbin Wang

**Affiliations:** Department of Veterinary Surgery, College of Veterinary Medicine, Northeast Agricultural University, Harbin 150030, China; liuboyang1994neau@163.com (B.L.); gam2006gx@163.com (X.G.); junema0420@126.com (J.M.); jiaozhihui1993neau@163.com (Z.J.); jhxiao1970@163.com (J.X.)

**Keywords:** *Culex tritaeniorhynchus*, Japanese encephalitis, host, environmental factors, maximum entropy model, mosquito-borne zoonosis

## Abstract

*Culex tritaeniorhynchus* is an important vector that transmits a variety of human and animal diseases. Japanese encephalitis (JE), an endemic disease in the Asia-Pacific region, is primarily transmitted by *Cx. tritaeniorhynchus*. Insufficient monitoring of vector mosquitoes has led to a poor understanding of the distribution of *Cx. tritaeniorhynchus* in China. To delineate the habitat of *Cx. tritaeniorhynchus* and any host and environmental factors that affect its distribution, we used a maximum entropy modeling method to predict its distribution in China. Our models provided high resolution predictions on the potential distribution of *Cx. tritaeniorhynchus*. The predicted suitable habitats of the JE vector were correlated with areas of high JE incidence in parts of China. Factors driving the distribution of *Cx. tritaeniorhynchus* in China were also revealed by our models. Furthermore, human population density and the maximum NDVI were the most important predictors in our models. Bioclimate factors and elevation also significantly impacted the distribution of *Cx. tritaeniorhynchus*. Our findings may serve as a reference for vector and disease control.

## 1. Introduction

Japanese encephalitis (JE) is a mosquito-borne zoonosis caused by infection with Japanese encephalitis virus (JEV). JEV is a member of the genus *Flavivirus*, family *Flaviviridae* [1]. JE usually manifests as mild central nervous symptoms, primarily in children and adolescents [2,3]. A small number of cases cause serious viral encephalitis and the disease has a mortality rate of approximately 20% to 40% [4,5]. Permanent neuropsychiatric sequelae may occur in up to 50% of JE survivors with encephalitis symptoms [6]. JE is prevalent in most parts of Asia and the Western Pacific, and has been estimated to result in approximately 67,900 cases annually [7]. JEV also causes reproductive losses and encephalitis in animals, such as swine, equine and cattle [8,9]. Unlike other animal hosts, swine play an important role in the spread of JEV as the major amplifying host [10].

All provinces of China, except Xinjiang and Qinghai, have reported cases of JE. Since 1951, JE has been included on the list of notifiable infectious diseases class B in China. The first confirmed JE case in China was in 1949, and the highest morbidity (20.92/100,000) occurred in 1971 [11]. A national vaccination program against JE was implemented in the 1970s [12]. The attenuated vaccine SA 14-14-2 developed in China is now widely used in JE-endemic countries [13].

In Asia, the principle vector of JE is *Culex tritaeniorhynchus* [14]. *Cx. tritaeniorhynchus* is a mosquito species that is widespread in Asia, the Mediterranean and Afrotropical region [15]. In addition to JE, *Cx. tritaeniorhynchus* also has the ability to transmit some other human and animal viral diseases such as Rift Valley fever, West Nile fever, Dengue fever and Tembusu virus infection [16,17,18,19]. It is widely accepted that *Cx. tritaeniorhynchus* prefers low lying water bodies with abundant plants, so paddy fields are a major habitat of the larvae [20,21]. As the area for rice planting has expanded in the last 40 years, *Cx. tritaeniorhynchus* has gained a broader habitat in countries and regions with developed levels of agriculture [22,23]. According to FAO Statistical databases, China has the second biggest rice planting area (30,449,860 hectares in 2016) in the world and is facing a growing public health threat.

In recent years, ecological niche modeling techniques have demonstrated their outstanding ability to predict the spatial distribution of various species and epidemic diseases, such as snails in South Africa, *Toxoplasma gondii oocysts* in China, *Bacillus anthracis* in Zimbabwe and plague in the U.S [24,25,26,27]. This modeling relies on high quality and high resolution information about environmental layers for the research area, which can help to provide good predictions with limited occurrence data.

The factors that influence the distribution of *Cx. tritaeniorhynchus* in China remain unclear. In this study, we applied a maximum entropy (Maxent) niche modelling method to predict the potential suitable habitats of *Cx. tritaeniorhynchus* in China. By modeling the spatial distribution of this insect vector, we can identify the main areas under threat of JE proliferation and other vector-borne diseases. It is also possible to understand the specific role of environmental factors that drive the distribution of the JE vector in China.

## 2. Methods

### 2.1. Mosquito Presence Data

The *Cx. tritaeniorhynchus* larvae and adult collections in China, used in our research, were obtained by literature retrieval. Literatures which provided sufficient information on collection locations to confirm coordinates were adopted. The records were taken from the period between 1970 and 2015. Detailed sampling time and literature sources are given in Table S1. Google Earth software was used to acquire the coordinates of mosquito collection points in the provided locations, when coordinates were not given in the literature. To match the resolution (1 km × 1 km) of environmental layers used in this study, presence records within 1 km^2^ were considered as one point.

### 2.2. Environmental Variables and Data Processing

We obtained twenty-seven environmental variable layers in total, including bioclimate data, elevation data, vegetation index and host population densities (Table 1). Bioclimate variables were downloaded from the WorldClim dataset. Nineteen variables that reflect temperature and precipitation conditions for 1970–2000 were the latest available data. Elevation data was obtained from the NASA’s Shuttle Radar Topography Mission (SRTM) Digital Elevation Data. In addition to altitude, slope and aspect were also considered as geographical factors affecting mosquito habitats [28]. Slope represents the flow velocity and runoff rate of surface and subsurface water, which affects the abundance of surface water and the soil moisture content [29]. Aspect affects the exposure to sunlight, which has a direct effect on the growth of plants [30]. Slope and aspect layers were extracted from the digital elevation model (DEM) using ArcGIS 10.2 (ESRI Inc., Redlands, CA, USA). We considered the Normalized Difference Vegetation Index (NDVI) as a predictor that reflects vegetation coverage. The NDVI value has been widely accepted as an important predictor for the presence of mosquitoes. It has been referenced in mosquito predictive studies, such as identifying mosquito clusters in urban areas and predicting the peak production of mosquitoes in rice fields [31,32]. The maximum, average and minimum NDVI were derived from Moderate Resolution Imaging Spectroradiometer (MODIS) images captured by satellite Terra. Human population density data for China was provided by the Chinese Academy of Sciences. Pig density data for China was obtained from the Livestock Geo-Wiki [33].

All environmental layers were treated as follows: (1) resampled to the resolution of 1 km × 1 km; (2) defined projection to GCS_WGS_1984; (3) clipped to the geographical area of China; (4) converted to ASCII format. All operations above were accomplished in ArcGIS 10.2. A strong correlation among bioclimate variables usually exists in the modeling, which may unjustifiably affect results [34]. The possible collinearity amongst the variables was investigated by calculating the variance inflation factor (VIF), using the *car* package in R [35,36,37]. The principle we selected the variables was that the VIF value is less than 10 [38].

### 2.3. Establishment of Maxent Models

Maxent 3.4.1 (http://biodiversityinformatics.amnh.org/open_source/maxent/) was used to establish the distribution models of *Cx. tritaeniorhynchus*. Due to the lack of absence data, a presence-only modeling approach was adopted in our study. Compared with other modeling methods using presence-only data, Maxent showed outstanding predictive performance in species niches and distribution modeling, even when the sample size was very small [39,40,41]. 

We initially hypothesized that host population densities (human and pigs) may be strong predictors that may obscure the effect of other variables [42]. We ran the program with two different combinations of layers. Layers including host population densities were used in the first round of modeling, and layers excluding host population densities were used for the second round of modeling. We set the random test percentage as 25%, meaning that 75% of the points would be used for training and 25% for testing [43,44]. And we ran Maxent with 10 cross-validation replicates [45].

Sampling bias is a general problem in the modeling of species distribution [46]. Sampling locations are usually conveniently accessed and these locations do not reflect the real distribution of the target species. In the default setting, background points are randomly selected by Maxent software in the entire research area. In order to counteract this effect in our models, we selected background points with the same spatial bias as the presence points [47]. Based on presence points, a surface of sampling intensity was created using the Gaussian kernel density estimator tool in ArcGIS [48,49]. This bias file was input into Maxent, and the software weighted the bias file to create 10,000 background points.

### 2.4. Model Evaluation and Interpretation

Model accuracy can be evaluated by area under the curve (AUC). The receiver operating characteristic curve (ROC) was generated by the Maxent software. A completely random prediction leads to an AUC value of 0.5. If the AUC value is more than 0.5, it means that the prediction is better than random. The closer the AUC value is to 1, the better the prediction performance will be. 

A jackknife test, built in Maxent software, was selected to assess the importance of each variable in the model [50]. The jackknife test is to drop each variable in turn in each run, and then use only each variable in turn in each run. Whether the training gain decreased when the variable was dropped or increased when used alone, this variable can be thought as containing information that no other variables have. This shows the importance of each variable to the model. We can also refer to the “Percent contribution” given by Maxent to judge the relative contribution of each variable to the model. Response curves provided by Maxent represent different models, which were created by using only the corresponding variable. Each curve shows the trend of predictive suitability as the variable varies. This can be interpreted as the specific impact of variables on predictive suitability.

## 3. Results

### 3.1. Selection of Variables

By adopting a one-by-one elimination method, variables with VIF > 10 were removed out of the final model. Six variables (Bio 3, Bio 5, Bio 11, Bio 14, Bio 15 and Bio 18) were selected from nineteen bioclimate variables.

### 3.2. Cx. tritaeniorhynchus Habitat Suitability

**Figure 1:**
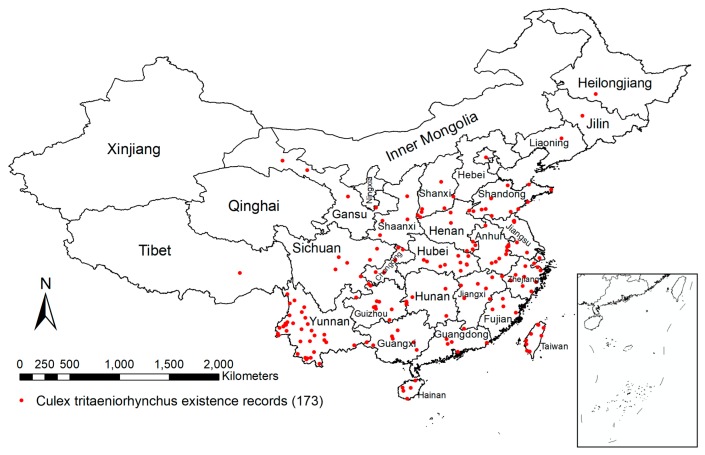
*Cx. tritaeniorhynchus* existence records. *Cx. tritaeniorhynchus* existence records in China collected by literature retrieval.

**Figure 2:**
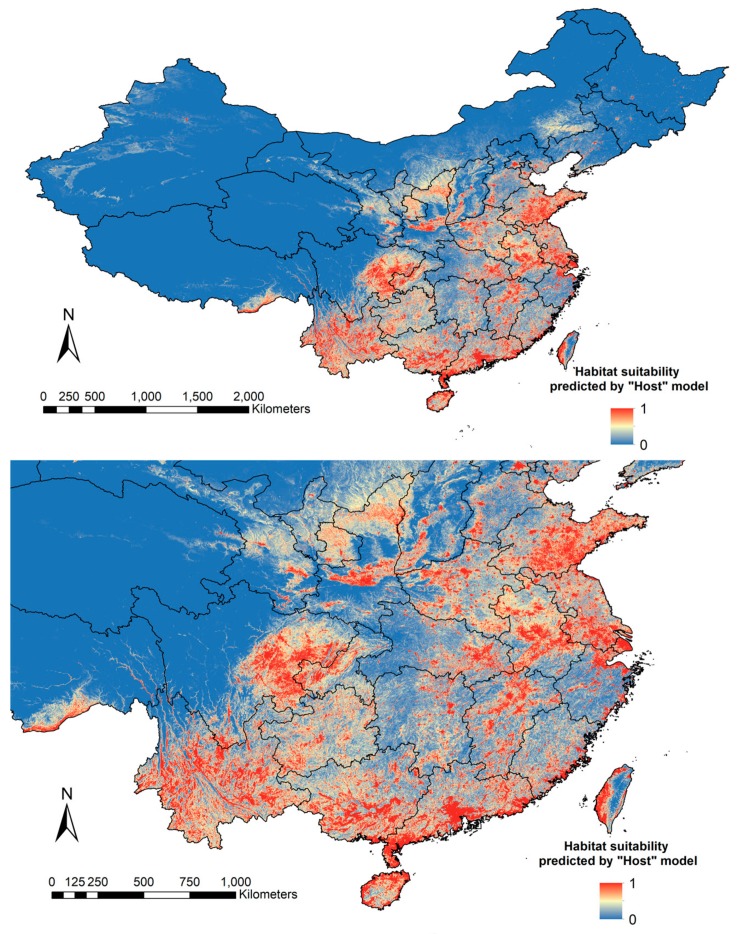
Distribution prediction map of *Cx. tritaeniorhynchus* based on the “Host” model. Prediction of habitat suitability of *Cx. tritaeniorhynchus* by using layers including human population density and pig density (the “Host” model, *n* = 14).

**Figure 3:**
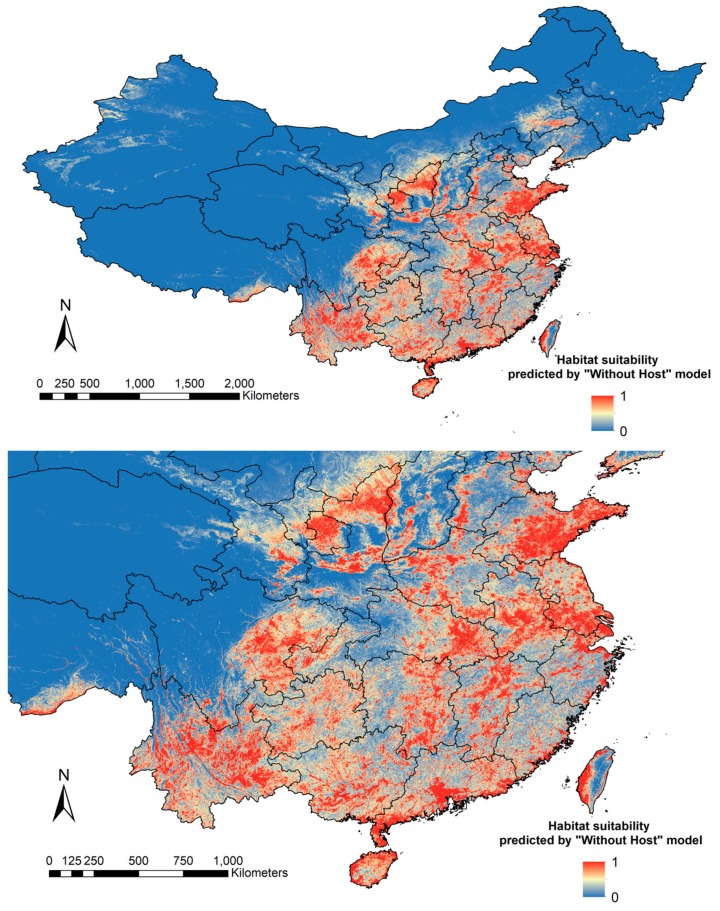
Distribution prediction map of *Cx. tritaeniorhynchus* based on the “Without Host” model. Prediction of habitat suitability of *Cx. tritaeniorhynchus* by using layers excluding human population density and pig density (the “Without Host” model, *n* = 12).

A total of 173 *Cx. tritaeniorhynchus* collection points were counted in our models (Figure 1). The distribution prediction maps for *Cx. tritaeniorhynchus* are shown in Figure 2 and Figure 3.

We found that both models predicted similar geographical areas as having a medium to high probability for the presence of *Cx. tritaeniorhynchus*. In central China, southern Shaanxi and Shanxi showed high habitat suitability of *Cx. tritaeniorhynchus*. In eastern China, extensive areas in Henan and Anhui, and southeast Shandong showed high probabilities of *Cx. tritaeniorhynchus* presence, as well as the Yangtze River Delta area. In the southwest of China, Sichuan, Chongqing, Yunnan and Guizhou provinces were the most likely areas for the presence of *Cx. tritaeniorhynchus*. Southern Guangxi and Guangdong, and coastal areas of Hainan, Fujian and Taiwan can also be regarded as suitable habitats. It should be noted that both models gave a high prediction for a limited border area in southeast Tibet. The two models showed some disagreements concerning the central and eastern region of China. The model including host population densities gave a prediction of medium probability for eastern Gansu, northern Shaanxi, eastern Hubei and Hunan, whereas the “Without Host” model gave these areas a higher probability. The prediction of “Without Host” model for southeast Shandong, northern Jiangsu and Jiangxi was broader and higher than that of the “Host” model. In general, the “Host” model provided a prediction of more conservative in extent and lower suitability of *Cx. tritaeniorhynchus* habitats in China.

### 3.3. Model Evaluation

The model evaluation indicators are shown in Table 2. Both models showed good performance. The model including host population densities gave higher training AUC and test AUC. Compared to the “Host” model, the “Without Host” model predicted a significantly broader exposed area, but a slightly larger exposed population. This indicated that more sparsely populated area was predicted as suitable habitat by the “Without Host” model. Sensitivity equals specificity threshold was applied to generate binary models and calculate test omission rate [41,47,51,52]. Binary models representing suitable/unsuitable were shown in Supplementary File S1.

### 3.4. Importance and Contribution of Variables

**Figure 4:**
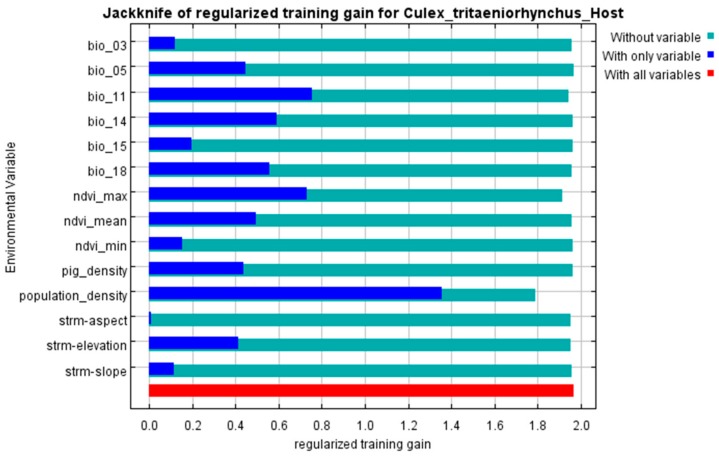
Results of the jackknife test for the “Host” model. A longer blue bar indicates a greater importance of that variable when it was used alone. A shorter green bar indicates a greater reduction in training gain when that variable was omitted.

**Figure 5:**
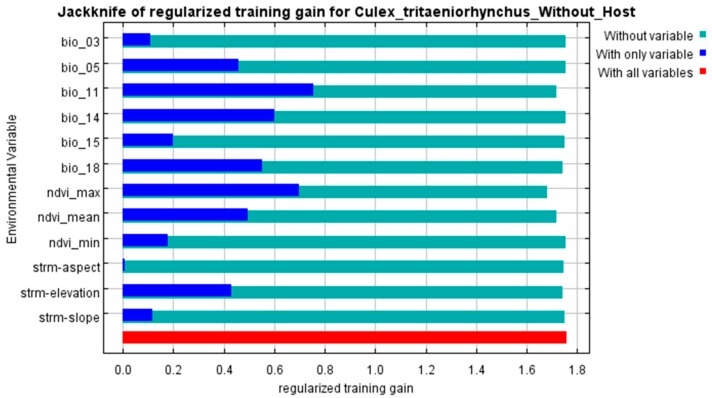
Results of the jackknife test for the “Without Host” model. A longer blue bar indicates a greater importance of that variable when it was used alone. A shorter green bar indicates a greater reduction in training gain when that variable was omitted.

According to the jackknife test (Figure 4 and Figure 5), it was obvious that human population density shows the greatest importance to the “Host” model. The increase in training gain when population density was used alone and the reduction when it was discarded were both the largest. In addition to population density, Bio 5, Bio 11, Bio 14, Bio 18, NDVI max, pig density and elevation also showed significant importance to the prediction.

The percent contribution of each variable in the two models are shown in Table 3. Bio 5, Bio11, Bio 14, Bio 18, NDVI max and elevation were regarded as variables with greater contributions in both models. In the “Host” model, human population density had the greatest relative contribution with 66.20%, whereas pig density was 0.41%. Maximum NDVI had the highest contribution (35.28%) in the “Without Host” model, followed by Bio 14 (32.21%).

### 3.5. Response Curves of Variables

**Figure 6:**
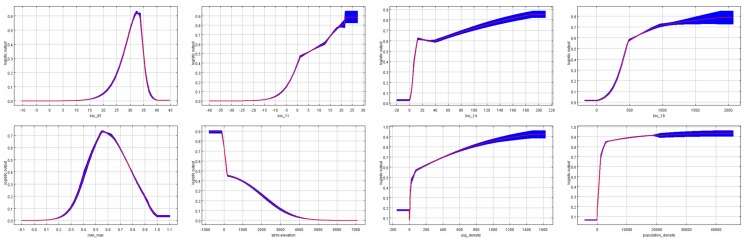
Response curves for representative variables.

According to their contribution and importance to the model, response curves of representative variables are shown in Figure 6.

## 4. Discussion

The two models in our study gave similar predictions of the distribution of the JE vector *Cx. tritaeniorhynchus* in China. The highly suitable habitats were mainly in the southwestern, central, eastern, and coastal areas of China. This coincides with the spatial distribution of JE in China. Since the 21st century, the southwestern region has experienced the most serious JE epidemics in China. According to the China CDC, the average incidence of JE in Guizhou (2.36/100,000), Chongqing (1.28/100,000), Sichuan (1.02/100,000), and Yunnan (0.91/100,000) ranked in the top four in China, far above the national average (0.37/100,000). Shaanxi (0.61/100,000), Henan (0.52/100,000) and Anhui (0.40/100,000) also had a high incidence of JE, ranking fifth to seventh. In recent years, due to advanced economic levels and higher immunization coverage in the coastal areas of China, the incidence of JE has been relatively low. In the 1970s, areas such as Shandong, Jiangsu, Zhejiang, Guangdong and Guangxi were once the most serious epidemic areas of JE in China [53]. There are certainly abundant vectors in areas that experience JE epidemics, which shows that our predictions are relatively accurate.

The predictions were also supported by reports of JE epidemics and the isolation of JEV in recent years. Human JE cases were obtained from literature reports and ProMED mail (http://www.promedmail.org/) [2,5,54,55,56,57,58,59]. The reported locations were mostly located in the high-risk areas predicted by our models (Table S2). In addition, there were several reports of JEV infection in pigs. JEV infections in swine were reported in Jiangsu (2008–2009), Shanxi (2009) and Sichuan (2012), places that were also predicted as high suitability habitats [60,61,62]. However, due to the limited numbers of JE cases obtained, this is not sufficient for demonstrating the predictive power of the model. Furthermore, some additional mosquito species within the genus *Culex* and *Aedes* can also transmit JEV in some regions [63,64,65]. Therefore, some of the observed JE cases may not be due to *Cx. tritaeniorhynchus* solely. More detailed information on JE cases and future mosquito surveys for suspected high-risk areas are required to make a thorough validation of our model’s predictive accuracy.

The “Host” model was more conservative whereas the “Without Host” model predicted a more extensive distribution of high-risk areas. Unsurprisingly, human activities led to such a difference. Habitat suitability may be affected by human interventions. According to the response curve, the habitat suitability rose sharply with the increase of population density (Figure 6). The most obvious impact of human activities on the distribution of mosquitoes is that mosquitoes have an abundant food source (blood) in densely populated areas. Furthermore, manmade structures not only provide shelter for humans, but also provide mosquitoes with a suitable living environment. As expected, the density of pigs also has an impact on the distribution of *Cx. tritaeniorhynchus*, but was not found to be as high as that of human influence.

Bioclimate variables played an important role in the modeling. The curve of Bio 5 (maximum temperature of the warmest month) rose first and then dropped, and the peak was at 32.5 °C. This shows that mosquitoes are benefited from higher temperatures, however, excessive temperatures have a negative effect on their survival. In another study, the upper limit of the suitable temperature for JE vectors was considered to be 34.5 °C in India [66]. At higher temperatures, mosquito bites become inactive and the mortality rate increases [67]. Bio 11 (mean temperature of the coldest month) represents the resistance of *Cx. tritaeniorhynchus* to low temperatures. Although there is evidence that adult *Cx. tritaeniorhynchus* can overwinter [68], their activity and number in winter were significantly lower. Only 10 female *Cx. tritaeniorhynchus* were collected from January to April in 2008 in a park in Tokyo, while 14,069 females were collected from April to November in 2007 [69]. The cold winter in northeast China is challenging for the mosquito survival and activity. Bio 14 (precipitation of the driest month) showed the adaptability of mosquitoes to drought. It is known that adequate precipitation is strictly required by *Cx. tritaeniorhynchus* eggs to maintain activity and hatch [70]. However, Xinjiang is a typically arid and semiarid province in China [71]. Two vast deserts, the Taklimakan Desert and Gurbantunggut Desert, are located in Xinjiang and occupy most of Xinjiang. The lack of precipitation makes the survival of mosquitoes difficult and this explains why there have never been JE cases in Xinjiang. Bio 18 (precipitation of the warmest quarter) demonstrated that summers with abundant rainfall promote the reproduction of mosquitoes.

The maximum NDVI was the most powerful predictor in the “Without Host” model. Vegetation coverage helps to avoid high temperatures and rapid evaporation of surface water caused by direct exposure to strong sunlight. Blood is sucked by female mosquitoes for reproduction, while both males and females obtain energy by feeding on sugar [72]. Mosquitoes get sugar supplements mainly from fruits and nectar [73]. Therefore, vegetation coverage provides not only suitable resting places, but also a sufficient source of sugar for mosquitoes. However, according to the response curve, when the NDVI value was too high (0.55), the suitability began to decline. As observed in a previous study, lush rice led to a dramatic decrease in the number of mosquito larvae [74]. Vegetation that is too dense will limit the sunlight received and cause temperatures to be too low. 

Elevation was also considered to be an important factor that affects the distribution of *Cx. tritaeniorhynchus* in both models. The response curve dropped rapidly as elevation rose. Low temperatures and thin air at high altitudes lead to sparse blood meal hosts and plants [75]. Low lying terrain is also beneficial for the accumulation of water. In a mosquito survey in Yunnan province, the number of *Cx. tritaeniorhynchus* decreased rapidly with elevation, and at an altitude of more than 3000 m no mosquitoes were captured [76]. The high predictive suitability of low altitude regions was reflected in two typical basins in our models, the Sichuan Basin and the Ordos Basin. In fact, the incidence of JE in these two areas is indeed high. On the contrary, Qinghai (free of JE) and Tibet (few JE cases) benefit from the existence of the Qinghai-Tibet Plateau, the highest plateau in the world.

Two areas require particular attention. A small border area in southeast Tibet was predicted to have a high habitat suitability. In this area, JEV isolates were reported in 2009, indicating that local vector and virus surveillance is an important requirement [77]. The surrounding area of the Taklimakan Desert in southern Xinjiang and the border areas of northern Xinjiang have the possibility of establishing habitat for *Cx. tritaeniorhynchus*.

Our research is novel in our focus on modeling the distribution of *Cx. tritaeniorhynchus* for the entire territory of China, with consideration for vegetation and host factors. A previous work mapped the distribution of *Cx. tritaeniorhynchus* within JE risk areas [78]. However, Tibet, Xinjiang and Qinghai province were not taken into consideration in this study. Human JE cases have been reported in Tibet since 2006, according to the China CDC (http://www.Chinacdc.cn/en/). And JEV isolates (from mosquitoes, pigs and humans) have been described in Tibet [77,79,80]. This demonstrated that JEV is currently circulating in Tibet, so we think it is necessary to include Tibet in the modeling. Xinjiang and Qinghai should also be considered to provide early warning of possible future JE invasion. Our research also reveals the relationship between habitat preference of *Cx. tritaeniorhynchus* and environmental factors. And the jackknife test and response curves are helpful to understand the conditions that affect the survival of mosquitoes. Compared with the variables used in the previous research (land surface temperature and tasselled cap wetness), we believe that the variables adopted in our research (Bioclimate variables) can better reflect the climate seasonality and complexity, especially for a country with a large territory like China, which crossed several climate zones. In addition, the prediction of the previous model may not be accurate in some areas. For example, there were records of *Cx. tritaeniorhynchus* in Hainan (Figure 1) but the previous prediction for Hainan was extreme low. Sampling bias was also treated reasonably in our model. Based on these efforts, we obtained a more reliable and precise prediction result for China and discovered potential habitats in Xinjiang and Tibet. Furthermore, our study is unique in its purpose of exploring the effects of hosts and environment on *Cx. tritaeniorhynchus* distribution. Host factors (densities of humans and pigs) were introduced into our models. By establishing the “Host” model, we investigated the effects of the existence of hosts on mosquitoes. Similarly, by establishing the “Without Host” model, we obtained the true impact of environmental variables on the distribution of *Cx. tritaeniorhynchus* and defined all possible suitable mosquito habitats for breeding, resting, and foraging activities. The results have important significance for guiding the targeted prevention and control of JE in China. Immunization should be promoted for susceptible human and livestock populations in the high-risk areas predicted by our models. The “Without Host” model gave a broader range of suitable areas for the *Cx. tritaeniorhynchus* survival, including areas that are sparsely populated at present. Possible future population migration needs to take this into account. At the same time, *Cx. tritaeniorhynchus* was also proved to be the vector for other zoonoses, such as Rift Valley fever, West Nile fever and Dengue fever. Pathogen surveillance in vectors of these diseases is necessary, especially as RVF and WNV have not yet been reported in China. These two exotic animal diseases have been listed in the Medium and Long-term State Strategy of Animal Disease Prevention and Control of China.

A mark-release-recapture study has shown the ability of *Cx. tritaeniorhynchus* to disperse more than 5 km, which may cause additional errors with predictions at the 1 km × 1 km resolution [81]. Due to many factors, it is difficult to completely randomize the experimental sampling sites. As with other presence-only modeling approaches, sampling bias is an unavoidable problem. Although we have performed a selection of background points to reduce the influence of sampling bias on the prediction results, the predictions should be interpreted with caution and need to be corroborated by mosquito surveys [40,82].

Our current research on the impact of environmental factors on mosquito distribution is limited, as we only used data on overall climate and vegetation status. If more detailed data were to be introduced in a further study, such as monthly meteorological data, there would be a deeper understanding of the impact at different time-points.

## 5. Conclusions

In our current study, we modelled the spatial distribution of *Cx. tritaeniorhynchus* in China, based on the Maxent niche modelling method. The central, eastern, southwestern and coastal area of China were predicted to be suitable habitats for *Cx. tritaeniorhynchus*. The most powerful predictors were human population density and maximum NDVI. Several bioclimate factors (Bio 5, Bio 11, Bio 14 and Bio 18) and elevation also had significant impacts on the distribution of *Cx. tritaeniorhynchus*. Our research reveals the relationship between habitat preference of *Cx. tritaeniorhynchus* and environmental factors. The results have important implications for the prevention and control of JE and other vector-borne infectious diseases.

## Figures and Tables

**Table 1:** Variables used in the modeling.

Variable	Description	Date	Resolution	Source
Bio1	Annual mean temperature	1970–2000	30 arc sec	WorldClim ^a^
Bio2	Mean diurnal range	1970–2000	30 arc sec	WorldClim ^a^
Bio3	Isothermality	1970–2000	30 arc sec	WorldClim ^a^
Bio4	Temperature seasonality	1970–2000	30 arc sec	WorldClim ^a^
Bio5	Maximum temperature of the warmest month	1970–2000	30 arc sec	WorldClim ^a^
Bio6	Minimum temperature of the coldest month	1970–2000	30 arc sec	WorldClim ^a^
Bio7	Temperature annual range	1970–2000	30 arc sec	WorldClim ^a^
Bio8	Mean temperature of the wettest quarter	1970–2000	30 arc sec	WorldClim ^a^
Bio9	Mean temperature of the driest quarter	1970–2000	30 arc sec	WorldClim ^a^
Bio10	Mean temperature of the warmest quarter	1970–2000	30 arc sec	WorldClim ^a^
Bio11	Mean temperature of the coldest quarter	1970–2000	30 arc sec	WorldClim ^a^
Bio12	Annual precipitation	1970–2000	30 arc sec	WorldClim ^a^
Bio13	Precipitation of the wettest month	1970–2000	30 arc sec	WorldClim ^a^
Bio14	Precipitation of the driest month	1970–2000	30 arc sec	WorldClim ^a^
Bio15	Precipitation seasonality	1970–2000	30 arc sec	WorldClim ^a^
Bio16	Precipitation of the wettest quarter	1970–2000	30 arc sec	WorldClim ^a^
Bio17	Precipitation of the driest quarter	1970–2000	30 arc sec	WorldClim ^a^
Bio18	Precipitation of the warmest quarter	1970–2000	30 arc sec	WorldClim ^a^
Bio19	Precipitation of the coldest quarter	1970–2000	30 arc sec	WorldClim ^a^
NDVIMAX	Maximum NDVI	2010	250 m	MODIS ^b^
NDVIMIN	Minimum NDVI	2010	250 m	MODIS ^b^
NDVIMEAN	Average NDVI	2010	250 m	MODIS ^b^
Elevation	Elevation above sea level	2017	250 m	STRM ^c^
Slope	Slope	2017	250 m	STRM ^c^
Aspect	Aspect ratio	2017	250 m	STRM ^c^
Human	Human population density	2010	1 km	CAS ^d^
Pig	Pig density	2006	1 km	Geo-Wiki ^e^

^a^ WorldClim Global Climate database version 2, available at: http://worldclim.org/version2; ^b^ Moderate Resolution Imaging Spectrometer (MODIS), available at: https://lpdaac.usgs.gov/; ^c^ Shuttle Radar Topography Mission (SRTM) 250 m digital elevation data version 4.1, available at: http://srtm.csi.cgiar.org//; ^d^ Human population density grid data of China, obtained from the Chinese Academy of Sciences (CAS); ^e^ Global Pig density grid data, available at: http://www.livestock.geo-wiki.org.

**Table 2:** Model evaluation indicators.

Model	Variables	Training AUC	Test AUC	Standard Deviation	Threshold	Test Omission Rate	Exposed Area (Million km^2^)	Exposed Population (Million)
**Host**	14	0.967	0.950	0.012	0.169	0.174	1.237	670.165
**Without Host**	12	0.957	0.942	0.026	0.196	0.174	1.523	681.775

**Table 3:** The percent contribution of each variable in two models.

Variables	Contribution in “Host” Model	Contribution in “Without Host” Model	Rank in “Host” Model	Rank in “Without Host” Model
Bio 03	1.33	0.47	7	10
Bio 05	0.61	0.84	8	8
Bio 11	4.53	3.56	4	5
Bio 14	10.58	32.21	2	2
Bio 15	0.48	0.55	10	9
Bio 18	2.83	12.85	5	3
NDVI MAX	10.31	35.28	3	1
NDVI MEAN	0.47	2.61	11	6
NDVI MIN	0.10	0.09	14	12
Elevation	1.42	9.82	6	4
Slope	0.17	1.39	13	7
Aspect	0.57	0.33	9	11
Human density	66.20		1	
Pig density	0.41		12	

## Supplementary Materials

The following are available online at http://www.mdpi.com/1660-4601/15/9/1848/s1, Table S1: Literature Sources. Literature sources of mosquito collections, Table S2: Model predictions of reported human JE cases, Supplementary File S1: Binary models representing suitable/unsuitable areas.

## References

[1] Rice C.M. (1996). Flaviviridae: The viruses and their replication. Fields Virol..

[2] Zhang J.-S., Zhao Q.-M., Guo X.-F., Zuo S.-Q., Cheng J.-X., Jia N., Wu C., Dai P.-F., Zhao J.-Y. (2011). Isolation and genetic characteristics of human genotype 1 japanese encephalitis virus, China, 2009. PLoS ONE.

[3] Tao Z., Liu G., Wang M., Wang H., Lin X., Song L., Wang S., Wang H., Liu X., Cui N. (2014). Molecular epidemiology of japanese encephalitis virus in mosquitoes during an outbreak in China, 2013. Sci. Rep..

[4] Solomon T. (2006). Control of japanese encephalitis—Within our grasp?. N. Engl. J. Med..

[5] Tian H.-Y., Bi P., Cazelles B., Zhou S., Huang S.-Q., Yang J., Pei Y., Wu X.-X., Fu S.-H., Tong S.-L. (2015). How environmental conditions impact mosquito ecology and japanese encephalitis: An eco-epidemiological approach. Environ. Int..

[6] Ghosh D., Basu A. (2009). Japanese encephalitis—A pathological and clinical perspective. PLoS Negl. Trop. Dis.

[7] Campbell G.L., Hills S.L., Fischer M., Jacobson J.A., Hoke C.H., Hombach J.M., Marfin A.A., Solomon T., Tsai T.F., Tsu V.D. (2011). Estimated global incidence of Japanese encephalitis: A systematic review. Bull. WHO.

[8] Begum S., Sekar M., Gunaseelan L., Devi M. (2017). Detection of Japanese encephalitis viral antibodies from swine. Pharma Innov..

[9] Scherer W., Buescher E., Flemings M., Noguchi A., Scanlon J. (1959). Ecologic studies of Japanese encephalitis virus in japan. Am. J. Trop. Med. Hyg..

[10] Ricklin M.E., Garcìa-Nicolàs O., Brechbühl D., Python S., Zumkehr B., Posthaus H., Oevermann A., Summerfield A. (2016). Japanese encephalitis virus tropism in experimentally infected pigs. Vet. Res..

[11] Wang X., Lu Y., Zhang Y.-P., Chen Y., Liang X. (2004). Dynamic tendency of Japanese B encephalitis in China. Chin. J. Vaccines Immunizat..

[12] Gao X., Nasci R., Liang G. (2010). The neglected arboviral infections in mainland China. PLoS Negl. Trop. Dis.

[13] Zhang S., Yin Z., Suraratdecha C., Liu X., Li Y., Hills S., Zhang K., Chen Y., Liang X. (2011). Knowledge, attitudes and practices of caregivers regarding Japanese encephalitis in Shaanxi province, China. Public Health.

[14] Liu K., Qian Y., Jung Y.S., Zhou B., Cao R., Shen T., Shao D., Wei J., Ma Z., Chen P. (2017). Mosgctl-7, a c-type lectin protein, mediates Japanese encephalitis virus infection in mosquitoes. J. Virol..

[15] Patsoula E., Beleri S., Vakali A., Pervanidou D., Tegos N., Nearchou A., Daskalakis D., Mourelatos S., Hadjichristodoulou C. (2017). Records of Aedes albopictus (skuse, 1894) (diptera; culicidae) and Culex tritaeniorhynchus (diptera; culicidae) expansion in areas in mainland greece and islands. Vector Borne Zoonotic Dis..

[16] Hayes C., Basit A., Bagar S., Akhter R. (1980). Vector competence of Culex tritaeniorhynchus (diptera: Culicidae) for west nile virus. J. Med. Entomol..

[17] Sallam M.F., Al Ahmed A.M., Abdel-Dayem M.S., Abdullah M.A. (2013). Ecological niche modeling and land cover risk areas for rift valley fever vector, Culex tritaeniorhynchus giles in Jazan, Saudi Arabia. PLoS ONE.

[18] Lytra I., Emmanouel N. (2014). Study of culex tritaeniorhynchus and species composition of mosquitoes in a rice field in Greece. Acta Trop..

[19] Platt G., Way H., Bowen E., Simpson D., Hill M., Kamath S., Bendell P., Heathcote O. (1975). Arbovirus infections in Sarawak, October 1968–February 1970 Tembusu and Sindbis virus isolations from mosquitoes. Ann. Trop. Med. Parasitol..

[20] Sucharit S., Surathin K., Shrestha S. (1989). Vectors of Japanese encephalitis virus (jev): Species complexes of the vectors. S Asian J. Trop. Med. Public Health.

[21] Gratz N. The impact of rice production on vector-borne disease problems in developing countries. Vector-Borne Disease Control in Humans through Rice Agro-Ecosystems Management.

[22] Miller R.H., Masuoka P., Klein T.A., Kim H.-C., Somer T., Grieco J. (2012). Ecological niche modeling to estimate the distribution of Japanese encephalitis virus in Asia. PLoS Negl. Trop. Dis.

[23] Keiser J., Maltese M.F., Erlanger T.E., Bos R., Tanner M., Singer B.H., Utzinger J. (2005). Effect of irrigated rice agriculture on Japanese encephalitis, including challenges and opportunities for integrated vector management. Acta Trop..

[24] Manyangadze T., Chimbari M.J., Gebreslasie M., Ceccato P., Mukaratirwa S. (2016). Modelling the spatial and seasonal distribution of suitable habitats of schistosomiasis intermediate host snails using maxent in ndumo area, Kwazulu-natal province, South Africa. Parasites Vectors.

[25] Gao X., Wang H., Wang H., Qin H., Xiao J. (2016). Land use and soil contamination with Toxoplasma gondii oocysts in urban areas. Sci.Total Environ..

[26] Chikerema S., Murwira A., Matope G., Pfukenyi D. (2013). Spatial modelling of Bacillus anthracis ecological niche in Zimbabwe. Prevent. Vet. Med..

[27] Holt A.C., Salkeld D.J., Fritz C.L., Tucker J.R., Gong P. (2009). Spatial analysis of plague in California: Niche modeling predictions of the current distribution and potential response to climate change. Int. J. Health Geogr..

[28] Sallam M.F., Xue R.-D., Pereira R.M., Koehler P.G. (2016). Ecological niche modeling of mosquito vectors of west nile virus in st. John’s county, Florida, USA. Parasites Vectors.

[29] Gorsevski P.V., Gessler P., Foltz R.B. Spatial prediction of landslide hazard using discriminant analysis and GIS. Proceedings of the GIS in the Rockies 2000 Conference and Workshop: Applications for the 21st Century.

[30] Mushinzimana E., Munga S., Minakawa N., Li L., Feng C.-c., Bian L., Kitron U., Schmidt C., Beck L., Zhou G. (2006). Landscape determinants and remote sensing of anopheline mosquito larval habitats in the western Kenya highlands. Malar. J..

[31] Brown H., Diuk-Wasser M., Andreadis T., Fish D. (2008). Remotely-sensed vegetation indices identify mosquito clusters of West Nile virus vectors in an urban landscape in the northeastern United States. Vector Borne Zoonotic Dis..

[32] Wood B.L., Beck L.R., Washino R.K., Palchick S.M., Sebesta P.D. (1991). Spectral and spatial characterization of rice field mosquito habitat. Int. J. Remot. Sens..

[33] Robinson T.P., Wint G.W., Conchedda G., Van Boeckel T.P., Ercoli V., Palamara E., Cinardi G., D’Aietti L., Hay S.I., Gilbert M. (2014). Mapping the global distribution of livestock. PLoS ONE.

[34] Li Q., Ren H., Zheng L., Cao W., Zhang A., Zhuang D., Lu L., Jiang H. (2017). Ecological niche modeling identifies fine-scale areas at high risk of dengue fever in the pearl river delta, China. Int. J. Environ. Res. Public Health.

[35] Pradhan P. (2016). Strengthening maxent modelling through screening of redundant explanatory bioclimatic variables with variance inflation factor analysis. Researcher.

[36] Olivero J., Toxopeus A.G., Skidmore A.K., Real R. (2016). Testing the efficacy of downscaling in species distribution modelling: A comparison between maxent and favourability function models. Anim. Biodivers. Conserv..

[37] Arnould J.P., Monk J., Ierodiaconou D., Hindell M.A., Semmens J., Hoskins A.J., Costa D.P., Abernathy K., Marshall G.J. (2015). Use of anthropogenic sea floor structures by Australian fur seals: Potential positive ecological impacts of marine industrial development?. PLoS ONE.

[38] Kubota Y., Shiono T., Kusumoto B. (2015). Role of climate and geohistorical factors in driving plant richness patterns and endemicity on the east asian continental islands. Ecography.

[39] Phillips S.J., Dudík M. (2008). Modeling of species distributions with maxent: New extensions and a comprehensive evaluation. Ecography.

[40] Wisz M.S., Hijmans R., Li J., Peterson A.T., Graham C., Guisan A. (2008). Effects of sample size on the performance of species distribution models. Divers. Distrib..

[41] Cunze S., Koch L.K., Kochmann J., Klimpel S. (2016). Aedes albopictus and Aedes japonicus-two invasive mosquito species with different temperature niches in europe. Parasites Vectors.

[42] Burkett-Cadena N.D., McClure C.J., Estep L.K., Eubanks M.D. (2013). Hosts or habitats: What drives the spatial distribution of mosquitoes?. Ecosphere.

[43] Phillips S.J. (2017). A Brief Tutorial on Maxent. http://biodiversityinformatics.amnh.org/open_source/maxent/.

[44] Acharya B.K., Cao C., Xu M., Khanal L., Naeem S., Pandit S. (2018). Present and future of dengue fever in nepal: Mapping climatic suitability by ecological niche model. Int. J. Environ. Res. Public Health.

[45] Sage K.M., Johnson T.L., Teglas M.B., Nieto N.C., Schwan T.G. (2017). Ecological niche modeling and distribution of ornithodoros hermsi associated with tick-borne relapsing fever in western north America. PLoS Negl. Trop. Dis.

[46] Kramer-Schadt S., Niedballa J., Pilgrim J.D., Schröder B., Lindenborn J., Reinfelder V., Stillfried M., Heckmann I., Scharf A.K., Augeri D.M. (2013). The importance of correcting for sampling bias in Maxent species distribution models. Divers. Distrib..

[47] Conley A.K., Fuller D.O., Haddad N., Hassan A.N., Gad A.M., Beier J.C. (2014). Modeling the distribution of the West Nile and Rift Valley fever vector Culex pipiens in arid and semi-arid regions of the middle east and North Africa. Parasites Vectors.

[48] Phillips S.J., Dudík M., Elith J., Graham C.H., Lehmann A., Leathwick J., Ferrier S. (2009). Sample selection bias and presence-only distribution models: Implications for background and pseudo-absence data. Ecol. Appl..

[49] Elith J., Kearney M., Phillips S. (2010). The art of modelling range-shifting species. Meth. Eco. Evol..

[50] Shcheglovitova M., Anderson R.P. (2013). Estimating optimal complexity for ecological niche models: A jackknife approach for species with small sample sizes. Ecol. Modell..

[51] Liu C., Berry P.M., Dawson T.P., Pearson R.G. (2005). Selecting thresholds of occurrence in the prediction of species distributions. Ecography.

[52] Liu C., White M., Newell G. (2013). Selecting thresholds for the prediction of species occurrence with presence-only data. J. Biogeogr..

[53] Gao X., Li X., Li M., Fu S., Wang H., Lu Z., Cao Y., He Y., Zhu W., Zhang T. (2014). Vaccine strategies for the control and prevention of Japanese encephalitis in mainland China, 1951–2011. PLoS Negl. Trop. Dis.

[54] Zhang D., Pan W., Wu R., Wu J. (2010). The epidemiological analysis of epidemic Japanese encephalitis in 2005–2009 years in Fujian province. CIin. J. Dis. Control. Prev..

[55] Wang L.-H., Fu S.-H., Wang H.-Y., Liang X.-F., Cheng J.-X., Jing H.-M., Cai G.-L., Li X.-W., Ze W.-Y., Lv X.-J. (2007). Japanese encephalitis outbreak, Yuncheng, China, 2006. Em. Infect. Dis..

[56] Xufang Y., Huanyu W., Shihong F., Xiaoyan G., Shuye Z., Chunting L., Minghua L., Yougang Z., Guodong L. (2010). Etiological spectrum of clinically diagnosed Japanese encephalitis cases reported in guizhou province, China, in 2006. J. Clin. Microbiol..

[57] Hu Q., Chen B., Zhu Z., Tian J., Zhou Y., Zhang X., Zheng X. (2013). Recurrence of Japanese encephalitis epidemic in Wuhan, China, 2009–2010. PLoS ONE.

[58] Fu Y., Li Z.-l., Wang C.-l. (2002). Investigation of an outbreak of Japanese B encephalitis in hainan provicne. China Trop. Med..

[59] Gao J., Deng X.Y., Pei-Shan L.U., Chen Y., Fu-Bao M.A. (2014). Analysis on epidemiology of Japanese type b encephalitis in Jiangsu province, 2007–2012. Chin. J. Dis. Control Prev..

[60] Chu H., Wu Z., Chen H., Li C., Guo X., Liu R., Wang G., Zhou M., Zhao T. (2017). Japanese encephalitis virus infection rate and detection of genotype i from Culex tritaeniorhynchus collected from Jiangsu, China. Vector Borne Zoonotic Dis..

[61] Cao Q.S., Li X.M., Zhu Q.Y., Wang D.D., Chen H.C., Qian P. (2011). Isolation and molecular characterization of genotype 1 Japanese encephalitis virus, sx09s-01, from pigs in China. Virol. J..

[62] Yuan L., Wu R., Liu H., Wen X., Huang X., Wen Y., Ma X., Yan Q., Huang Y., Zhao Q. (2016). Tissue tropism and molecular characterization of a Japanese encephalitis virus strain isolated from pigs in southwest China. Virus Res..

[63] Flohic G.L., Porphyre V., Barbazan P., Gonzalez J.P. (2013). Review of climate, landscape, and viral genetics as drivers of the Japanese encephalitis virus ecology. PLoS. Negl. Trop. Dis..

[64] Jin L., Li D. (2008). A recent survey of mosquito fauna in Guandong province, southern China, with a review of past records. Med. Vet. Entomol..

[65] Kanojia P. (2007). Ecological study on mosquito vectors of Japanese encephalitis virus in bellary district, karnataka. Indian J. Med. Res..

[66] Murty U.S., Rao M.S., Arunachalam N. (2010). The effects of climatic factors on the distribution and abundance of Japanese encephalitis vectors in Kurnool district of Andhra Pradesh, India. J. Vector Borne Dis..

[67] Wang L., Hu W., Magalhaes R.J.S., Bi P., Ding F., Sun H., Li S., Yin W., Wei L., Liu Q. (2014). The role of environmental factors in the spatial distribution of Japanese encephalitis in mainland China. Environ. Int..

[68] Nabeshima T., Loan H.T.K., Inoue S., Sumiyoshi M., Haruta Y., Nga P.T., Huoung V.T.Q., del Carmen Parquet M., Hasebe F., Morita K. (2009). Evidence of frequent introductions of Japanese encephalitis virus from south-east asia and continental east Asia to Japan. J. Gen. Virol..

[69] Tsuda Y., Kim K.S. (2008). Sudden autumnal appearance of adult culex tritaeniorhynchus (diptera: Culicidae) at a park in urban Tokyo: First field evidence for prediapause migration. J. Med. Entomol..

[70] Smith W.H., Cervantes E.P., Bos R. (1988). Vector-borne disease control in humans through rice agroecosystem management. Parasitol. Today.

[71] Zhang Q., Li J., Singh V.P., Bai Y. (2012). Spi-based evaluation of drought events in Xinjiang, China. Nat. Hazards.

[72] Foster W.A. (1995). Mosquito sugar feeding and reproductive energetics. Ann. Rev. Entomol..

[73] Gu W., Müller G., Schlein Y., Novak R.J., Beier J.C. (2011). Natural plant sugar sources of anopheles mosquitoes strongly impact malaria transmission potential. PLoS ONE.

[74] Mutero C.M., Blank H., Konradsen F., Hoek W.V.D. (2000). Water management for controlling the breeding of Anopheles mosquitoes in rice irrigation schemes in Kenya. Acta Trop..

[75] Tatem A.J., Hay S.I., Rogers D.J. (2006). Global traffic and disease vector dispersal. Proc. Nalt. Acad. Sci. USA.

[76] Sun X., Fu S., Gong Z., Ge J., Meng W., Feng Y., Wang J., Zhai Y., Wang H., Nasci R. (2009). Distribution of arboviruses and mosquitoes in northwestern Yunnan province, China. Vector Borne Zoonotic Dis..

[77] Li Y.-X., Li M.-H., Fu S.-H., Chen W.-X., Liu Q.-Y., Zhang H.-L., Da W., Hu S.-L., La Mu S.D., Bai J. (2011). Japanese encephalitis, Tibet, China. Em. Infect. Dis..

[78] Longbottom J., Browne A.J., Pigott D.M., Sinka M.E., Golding N., Hay S.I., Moyes C.L., Shearer F.M. (2017). Mapping the spatial distribution of the Japanese encephalitis vector, Culex tritaeniorhynchus Giles, 1901 (diptera: Culicidae) within areas of Japanese encephalitis risk. Parasites Vectors.

[79] Li M.H., Fu S.H., Chen W.X., Wang H.Y., Cao Y.X., Liang G.D. (2014). Molecular characterization of full-length genome of Japanese encephalitis virus genotype v isolated from Tibet, China. Biomed. Environ. Sci..

[80] Zhang H., Rehman M.U., Li K., Luo H., Lan Y., Nabi F., Zhang L., Iqbal M.K., Zhu S., Javed M.T. (2017). Epidemiologic survey of Japanese encephalitis virus infection, Tibet, China, 2015. Emerg. Infect. Dis..

[81] Reisen W., Aslam Y., Siddiqui T., Khan A. (1978). A mark-release-recapture experiment with Culex tritaeniorhynchus Giles. Trans. R. Soc. Trop. Med. Hyg..

[82] Elith J., Phillips S.J., Hastie T., Dudík M., Chee Y.E., Yates C.J. (2011). A statistical explanation of maxent for ecologists. Divers. Distrib..

